# Factors Associated with Ineffectiveness of Sildenafil Treatment in Patients with End-Stage Heart Failure and Elevated Pulmonary Vascular Resistance

**DOI:** 10.3390/jcm9113539

**Published:** 2020-11-02

**Authors:** Wioletta Szczurek, Mariusz Gąsior, Michał Skrzypek, Ewa Romuk, Bożena Szyguła-Jurkiewicz

**Affiliations:** 1 Silesian Center for Heart Diseases in Zabrze, 41-800 Zabrze, Poland; 2 3rd Department of Cardiology, School of Medical Sciences in Zabrze, Medical University of Silesia, 40-055 Katowice, Poland; mgasior@op.pl (M.G.); centrala4@wp.pl (B.S.-J.); 3 Department of Biostatistics, School of Public Health in Bytom, Medical University of Silesia, 40-055 Katowice, Poland; mskrzypek@sum.edu.pl; 4 Department of Biochemistry, School of Medicine with the Division of Dentistry, Medical University of Silesia, 41-800 Zabrze, Poland; eromuk@gmail.com

**Keywords:** end-stage heart failure, pulmonary vascular resistance, sildenafil, factor

## Abstract

Introduction: Elevated pulmonary vascular resistance (PVR) unresponsive to vasodilator treatment is a marker of heart failure (HF) severity, and an important predictor of poor results of heart transplantation (HT). Objective: We sought to analyze factors associated with ineffectiveness of sildenafil treatment in end-stage HF patients with elevated PVR with particular emphasis placed on tenascin-C (TNC) serum concentrations. Patients and Methods: The study is an analysis of 132 end-stage HF patients referred for HT evaluation in the Cardiology Department between 2015 and 2018. TNC was measured by sandwich enzyme-linked immunosorbent assay (Human TNC, SunRedBio Technology, Shanghai, China). The endpoint was PVR > 3 Wood units after the six-month sildenafil therapy. Results: The median age was 58 years, and 90.2% were men. PVR >3 Wood units after 6 months of sildenafil treatment were found in 36.6% patients. The multivariable logistic regression analysis confirmed that TNC (OR = 1.004 (1.002–1.006), *p* = 0.0003), fibrinogen (OR= 1.019 (1.005–1.033), *p* = 0.085), creatinine (OR =1.025 (1.004–1.047), *p* = 0.0223) and right ventricular end-diastolic dimension (RVEDd) (OR = 1.279 (1.074–1.525), *p* = 0.0059) were independently associated with resistance to sildenafil treatment. Area under the ROC curves indicated an acceptable power of TNC (0.9680 (0.9444–0.9916)), fibrinogen (0.8187 (0.7456–0.8917)) and RVEDd (0.7577 (0.6723–0.8431)), as well as poor strength of creatinine (0.6025 (0.4981–0.7070)) for ineffectiveness of sildenafil treatment. Conclusions: Higher concentrations of TNC, fibrinogen and creatinine, as well as a larger RVEDd are independently associated with the ineffectiveness of sildenafil treatment. TNC has the strongest predictive power, sensitivity and specificity for evaluation of resistance to sildenafil treatment.

## 1. Introduction

Pulmonary hypertension (PH) secondary to the left ventricular dysfunction (group 2 PH) is the most common form of PH in clinical practice. This kind of PH is predominantly a consequence of elevated left atrial pressure transmitted backwards into pulmonary circulation [1]. However, structural remodeling and endothelial dysfunction in the pulmonary vascular tree result in an increase of pulmonary vascular resistance (PVR) in some heart failure (HF) patients, leading to severe PH [2,3]. Elevated PVR unresponsive to vasodilator treatment is a marker of disease severity, as well as an important predictor of poor results of heart transplantation (HT) [4]. The underlying cause of such an adverse outcome is the high risk of acute post-transplant right HF of the allograft suddenly exposed to vascular bed with elevated resistance [4].

According to the classification used by current guidelines, group 2 PH involves both passive and reactive components [1]. The elevation of left-sided filling pressures that results from left ventricular dysfunction leads to a passive increase in pulmonary venous pressure [3]. This passive component affects an endothelium-dependent reactive component and causes deficiency in nitric oxide-mediated vasodilation in the pulmonary arterial bed, as well as increased transpulmonary gradient and PVR [3]. The increase in pulmonary artery pressure at this stage of PH reflects pulmonary arterial vasoconstriction and pulmonary arteriolar and venous remodeling, as well as the elevation of pulmonary venous pressures. A long-term elevation of hydrostatic pressure induces neurohormonal activation and structural remodeling of the entire alveolar-capillary unit, leading to impaired gas diffusion [4]. In most patients, reactive pulmonary vasoconstriction may be reversed, if treated early enough and over a long period [5,6].

Standard pharmacological HF therapies involving decreasing left ventricular filling pressures affect only the passive component of type 2 PH [3,5]. Among therapies which impact the reactive component of PH, only phosphodiesterase-5 (PDE5) inhibitors decrease PVR, and do not increase left-sided filling pressures [3,5,7]. Previous analyses have demonstrated that the PDE5 inhibitor sildenafil significantly reduced pulmonary pressures and PVR in HF patients with group 2 PH [8,9,10,11,12,13]. Consequently, PDE5 inhibitors are frequently prescribed off-label in the patients with elevated PVR on standard optimal medical and device therapies [5,11,13].

It has been shown that at an early stage, secondary PH is reversible, but can gradually become irreversible due to the pulmonary vascular remodeling, thrombosis and vasoconstriction, as well as the structural and functional rebuilding of the extracellular matrix [14,15]. Pathological vascular and tissue remodeling is known to be accompanied by the re-occurrence of fetal variants of tenascin-C (TNC), an extracellular matrix glycoprotein, which is expressed only during the development of the embryonic heart and not in the normal adult heart [16,17,18]. Serum measurement of TNC level, because of its non-invasive nature, could be a potential clinical biomarker reflecting TNC expression [19].

Given the close relationship between TNC tissue and serum levels as well as vascular remodeling, we speculated that higher TNC serum concentrations might be associated with the presence of severe PH and no response to sildenafil treatment.

Therefore, we sought to analyze factors associated with the ineffectiveness of a six-month administration of sildenafil in patients who were considered high-risk candidates for HT because of the elevated PVR during baseline right heart catheterization (RHC), with particular emphasis placed on TNC serum concentrations. 

## 2. Materials and Methods

The study is an analysis of 132 end-stage HF patients (INTERMACS 4–6) referred for HT evaluation in the Cardiology Department between 2015 and 2018. The inclusion criteria were defined in the following way:

1. Severe PH defined as the presence of elevated systolic pulmonary artery pressure (sPAP ≥ 50 mmHg), the PVR > 3 Wood units or the transpulmonary gradient (TPG ≥ 15 mmHg) [1];
2. reversibility of elevated PVR during the use of nitroprusside infusion;
3. optimal medical and device therapy.

The exclusion criteria included: acute HF, HF due to valvular heart diseases, any previous valvular heart surgery, left ventricular assist device (LVAD) implantation, PH with irreversible PVR, neoplastic and autoimmune diseases, severe chronic obstructive pulmonary disease, history of pulmonary embolism, irreversible renal dysfunction (glomerular filtration rate < 30 mL/min/1.73 m^2^) and inotropic support at presentation, as well as the lack of right heart catheterization.

RHC was performed during the index hospitalization using a Swan–Ganz catheter. The cardiac output (CO) was measured by thermodilution using rapid bolus injection of 10 cc cold saline. In cases of severe tricuspid regurgitation, the CO was measured based on the estimates of oxygen uptake according to the Fick method. The cardiac index (CI) was calculated by dividing the CO obtained with either method by the body surface area (liters/min/m^2^). sPAP, diastolic PAP (dPAP) and mean PAP (mPAP), as well as mean pulmonary artery wedge pressure (mPCWP), were measured automatically. The transpulmonary gradient (TPG) was calculated as the difference between mPAP and mPCWP. PVR was calculated as the ratio of TPG to CO. After baseline hemodynamic data were acquired, all patients received an intravenous bolus infusion of nitroprusside in the starting dose of 10 ng/kg/min. The dose of nitroprusside was adjusted until reaching: 1. patient intolerance, 2. normalization in the values of PVR and TPG, 3. the reduction in systolic blood pressure above 85 mmHg. Hemodynamic measurements were repeated after five minutes. Patients were defined as responders to the vasodilator challenge if PVR decreased below 2.5 Wood units and systolic blood pressure did not drop below 85 mmHg. Reversibility of elevated PVR was demonstrated by the use of nitroprusside infusion in all patients, after which sildenafil was included at an initial dose of 3 × 25 mg, which was later escalated to the maximum tolerated dose. RHC was repeated 6 months after the initiation of sildenafil therapy in all patients.

Then, the population of patients was divided post hoc into two groups according to the presence or absence of severe PH (using the criteria of sPAP ≥ 50 mmHg and PVR > 3 Wood units and/or TPG ≥ 15 mmHg) after the six-month sildenafil treatment. Group A included the patients without severe PH, while group B contained the patient with severe PH despite the six-month sildenafil therapy.

The endpoint of the analysis was PVR > 3 Wood units after the six-month sildenafil therapy. 

### 2.1. Laboratory Testing

Blood samples were drawn after a 12-h fasting period at the baseline. The hematologic parameters of patients were analyzed using automated blood cell counters (Sysmex XS1000i and XE2100, Sysmex Corporation, Kobe, Japan). The intra-assay and inter-assay coefficients of variation of the blood samples were 5% and 4.5%, respectively. Hepatic and renal function parameters, cholesterol, triglycerides and albumin plasma concentrations were determined with a COBAS Integra 800 analyzer (Roche Instrument Center AG, Rotkreuz, Switzerland). Fibrinogen levels were measured in citrated plasma kept at −70 °C using a modified Clauss method (Dade Behring) and a Sysmex CA-6000 automated coagulation analyzer [10]. The mean value in our laboratory is 300 mg/dL, with a reference range of 200 to 400 mg/dL.

A highly sensitive latex-based immunoassay was used to detect plasma C-reactive protein with the Cobas Integra 70 analyzer (Roche Diagnostics, Ltd., Mannheim, Germany). The C-reactive protein levels were determined with a typical detection limit of 0.0175 mg/dL. Plasma concentration of N-terminal brain natriuretic peptide was measured by a commercially available kit from Roche Diagnostics (Mannheim, Germany) on an Elecsys 2010 analyzer with analytical sensitivity of <5 pg/mL. Human TNC was measured by sandwich enzyme-linked immunosorbent assay (ELISA) with the commercially available kit (Human Tenascin C ELISA, SunRedBio Technology Co, Ltd., Shanghai, China). The concentration of TNC was expressed as ng/L. The minimum detectable concentration for the TNC was 24.015 ng/L. Assay range was: 25 ng/L–6000 ng/L. This ELISA test was performed using BioTek Elx50 reader (BioTek Instruments Inc, Winooski, VT, USA, Tecan Group, Männedorf, Switzerland).

The study was conducted in compliance with the Ethics Committee of Medical University of Silesia (specific ethics code—KNW/0022/KB1/88/15) and in full compliance with the Declaration of Helsinki. A written, informed consent was obtained from all patients.

### 2.2. Statistical Analysis

The statistical analysis was performed using SAS software, version 9.4 (SAS Institute Inc., Cary, NC, USA). Continuous variables were expressed as a mean ± standard deviation if normally distributed, or as a median (25th–75th percentile) if skewed; categorical variables were expressed as percentages. Continuous variables were compared using the Student’s t-test or the Mann–Whitney test and categorical variables were compared using the chi-squared test. A univariable logistic regression analysis was performed to select the potential independent predictive factors of the end-point for inclusion in the multivariable analysis. The examined co-variables included laboratory parameters (albumin, total bilirubin, high-sensitivity C-reactive protein, fibrinogen, alkaline phosphatase (ALP), gamma glutamyl transpeptidase (GGTP), creatinine, uric acid, urea and TNC), and right ventricular end-diastolic dimension (RVEDD). Univariable predictors of the end-point with a *p*-value of <0.2 were entered into a multivariable logistic regression model with stepwise backward elimination. The correlation between explanatory variables was checked, and multicollinearity was evaluated by means of tolerance and Variance Inflation Factor (VIF). The C-statistic, deviance and Pearson goodness-of-fit statistics, as well as the Hosmer–Lemeshow test results for the final model were calculated. Differences were considered statistically significant at *p* < 0.05. The results were presented as odds ratios (ORs) with 95% confidence intervals (CIs). The receiver operating characteristic (ROC) curve was created to determine the utility of the factors obtained from multivariable logistic regression for the separation of the patients responding to the sildenafil therapy from those who do not respond to it. The optimal cut-off value for each factor was determined by using the Youden index. The prognostic strength of the factors was evaluated by the area under the curves (AUC) from the ROC analysis as well as their sensitivities and specificities. An AUC > 0.7 was considered clinically relevant.

## 3. Results

The median age of the patients was 58 (50–62) years, of whom 90.2% were men. In 51 (38%) of the included patients, severe tricuspid regurgitation was observed. All the included patients were classified as New York Heart Association (NYHA) classes III (32.6%) and IV (67.4%). Group A comprised 83 patients without severe PH. Group B comprised 49 patients considered high-risk for HT based on the presence of severe PH despite the six-month sildenafil therapy. All the patients were subjected to an optimal medical therapy and were on the same drug regimen that included: angiotensin converting enzyme (ACE) inhibitor (ramipril, 5–10 mg/d, or perindopril, 5–10 mf/d) or angiotensin receptor blocker (ARB) (2.3% of patients), beta-blockers (metoprolol CR, 100–150 mg/d, or carvedilol, 50–75 mg/d or bisoprolol, 5–7.5 mg/d), loop diuretics (furosemide, 120–160 mg/d or torasemide, 50–150 mg/d) and mineralocorticoid receptor antagonist (MRA) (spironolactone, 25–50 mg/d, epleronone 25–50 mg/d).

All patients were receiving implantable cardioverter defibrillator *(ICD) or* cardiac resynchronization *therapy with* defibrillator (CRT-D). There were no statistically significant differences in the mean dose of sildenafil in patients with PVR > 3 Wood units compared with patients with normal PVR after 6 months of sildenafil therapy (88.78 (19.80) vs. 92.77 (18.12); *p* = 0.24, respectively).

The baseline characteristics of the analyzed population at the time of inclusion to the study are summarized in Table 1.

Hemodynamic results of right heart catheterization at the baseline and after six months of sildenafil therapy are shown in Table 2.

According to the multivariable logistic regression analysis, TNC, fibrinogen and creatinine serum concentrations as well as the right ventricular end-diastolic diameter (RVEDd) were significantly associated with the ineffectiveness of sildenafil treatment in the analyzed group of patients (Table 3).

The ROC curve analysis has shown that TNC, fibrinogen and the RVEDd have an acceptable discriminatory power allowing for an effective separation of patients who respond to the sildenafil therapy from those who do not respond to it (Table 4).

**Figure 1:**
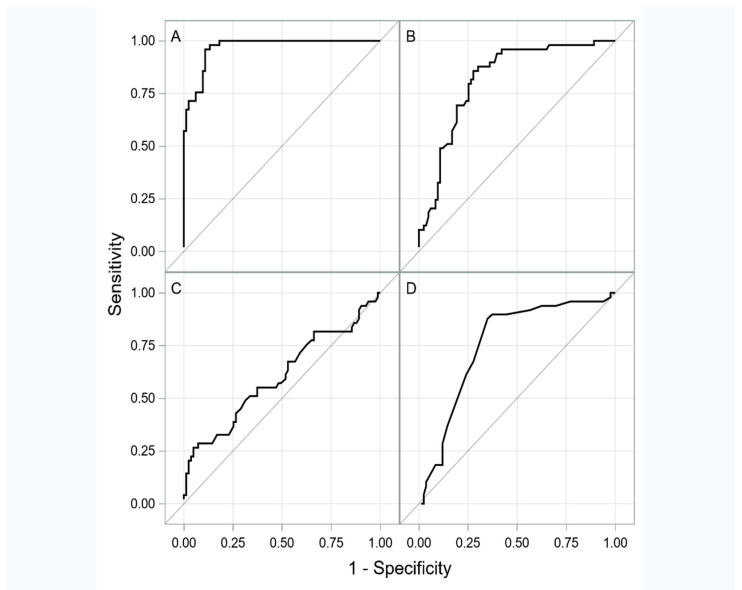
The ROC curves for tenascin C (**A**), fibrinogen (**B**), creatinine (**C**) and right ventricular end-diastolic dimension (**D**).

The results of the ROC curves analysis are shown in Figure 1A–D.

## 4. Discussion

Our single-center prospective study has shown for the first time that serum TNC levels were independently associated with the resistance to the sildenafil treatment during a six-month follow-up in patients with HF and elevated PVR. This is an important novel finding because fetal tenascin is functionally involved in tissue and vascular remodeling and, possibly, elevated levels of this glycoprotein may reflect advanced changes of the pulmonary vascular bed that do not respond to the sildenafil therapy. Fetal TNC has several functions that deteriorate cell adhesion, upregulate the activity of matrix metalloproteinases and their tissue inhibitors, cause the degradation of connective tissue, as well as stimulate the inflammatory response and the recruitment of myofibroblasts, and regulate the early stages of fibrosis. Furthermore, there is clear evidence that TNC promotes the proliferation of both pulmonary arterial smooth muscle cells and endothelial cells [20]. In addition, fetal TNC variants are functionally involved in the structural rebuilding of the cardiac extracellular matrix. Thus, it has been suggested that the re-expression of fetal TNC variants reflects the extent of cardiac and vascular remodeling as well as PH severity [20,21,22,23,24,25,26,27]. It is known that ventricular and vascular remodeling are of critical importance in the development of PH. Studies on animal models and clinical studies most clearly suggest that TNC plays a role not only of a prognostic and diagnostic factor, but also of a biomarker for therapeutic surveillance in PH [21,22,25]. In their study on experimental PH, Correira-Pinto et al. demonstrated the overexpression of TNC in the cardiac tissue, reflecting right ventricular pressure overload. Furthermore, TNC serum levels in this study correlated with sPAP and the right atrium area [22]. Additionally, the animal study conducted by Rabinovitch et al. demonstrated that pulmonary artery hypertrophy was associated with the TNC expression in arterial smooth muscle cells [23]. Moreover, the expression of TNC has been reported in the pulmonary arteries of animals with hypertensive remodeling [24]. In turn, Schermuly et al. demonstrated that the reversion of the remodeling process in chronic experimental PH after iloprost inhalation was reflected by the decrease in TNC occurrence [25]. Rohm et al. demonstrated in a clinical study that fetal TNC levels were increased in PH patients, compared with healthy controls [26]. Serum TNC concentrations in their research correlated with echocardiographic parameters such as the right atrium area and sPAP. Additionally, the authors have shown a significant correlation between TNC and B-type natriuretic peptide serum levels, as well as between TNC and the 6-min walk distance [26]. It has been discovered that patients with higher TNC serum concentrations had higher pulmonary arterial pressures and resistance, compared with those with lower values of this measure [27,28].

Another interesting finding of the present study was the strong and independent association between plasma fibrinogen levels and the ineffectiveness of sildenafil therapy in the analyzed group of patients. The mechanisms whereby elevated fibrinogen serum concentrations help to predict PH unresponsive to sildenafil treatment are still not fully understood and remain unclear. From the pathophysiological point of view, fibrinogen, which affects blood rheology, hemostasis, platelet aggregation, endothelial function and inflammation, can be associated with the development of PH. There is evidence that fibrinogen is the major determinant of plasma viscosity and hence the rheological properties of blood are affected by higher levels of plasma fibrinogen [29]. Furthermore, it is a natural blood constituent capable of inducing erythrocyte rouleaux formation. The hemorheologic consequences of hyperfibrinogenemia may limit the fluidity of blood and predispose one to thrombosis [30,31]. In addition, fibrinogen serves as the cross-link for glycoprotein IIb/IIIa on adjacent platelets and, therefore, it is an essential cofactor for aggregation. Platelets appear to be an integral part of the inflammatory process and may directly initiate an inflammatory response of the vessel wall [32]. There is evidence suggesting that both fibrinogen and its degradation products stimulate the vascular wall smooth muscle cells proliferation and migration, inhibit the synthesis of prostacycline in endothelial cells, and increase chemotaxis of leukocytes, monocytes and fibroblasts [33]. Furthermore, fibrinogen and its degradation products cause vasoconstriction through increased endothelin-1 release from pulmonary artery endothelial cells [34]. These mechanisms are closely related to development and pathogenesis of PH. Thus, higher levels of fibrinogen may reflect the severity of these processes in the pulmonary bed, and indirectly can identify patients who do not respond to sildenafil therapy.

We have also demonstrated the validity of another parameter associated with the ineffectiveness of sildenafil treatment, namely the higher creatinine concentration. The presence of kidney dysfunction is a key contributor to several pathophysiologic processes, which may amplify the deleterious effects of PH associated with left sided heart disease [35,36]. Several potential mechanisms may explain the high prevalence of kidney dysfunction in PH owing to left-sided heart disease [35,36,37,38] and its association with the lack of response to sildenafil treatment. One might speculate that a higher level of creatinine, reflecting a more advanced degree of kidney damage, indirectly indicates a more profound degree of vascular remodeling in other organs. There is also a strong correlation between elevated right atrial pressures and kidney function, suggesting the importance of venous congestion in kidney dysfunction [35,39,40]. Increased central venous pressures observed in type 2 PH are associated with the development of renal venous congestion and a gradual deterioration of renal function [36,37,38,39]. In turn, renal venous congestion affects the activation of the sympathetic nervous system and the renin-angiotensin-aldosterone system; it also induces the baroreceptor-associated, neural reflex and natriuretic peptide pathways. In addition, renal venous congestion causes endothelial changes from a quiescent redox profile to an activated pro-inflammatory, pro-oxidant and pro-vasoconstrictive state [40]. The activation of these mechanisms is closely related to the development and progression of PH, promoting a vicious cycle of unfavorable remodeling in the pulmonary and renal vascular walls. Furthermore, a reduction in the cardiac output secondary to the HF progress results in a disproportionate reduction in renal perfusion, which further leads to an increase in creatinine concentrations and a diminished glomerular filtration rate [38,39]. It seems that the combination of PH and kidney dysfunction contributes to adverse pulmonary remodeling, leading to the lack of response to sildenafil treatment. Our previous study also showed that the high modified Model for End-Stage Liver Disease (modMELD) score, reflecting liver and kidney dysfunction, may be a useful indicator of the ineffectiveness of sildenafil treatment in patients with end-stage HF evaluated for HT [35].

The right ventricular dimension in M-mode echocardiography was another independent noninvasive factor associated with the ineffectiveness of sildenafil treatment. Previous studies have shown that the right ventricular function is an important prognostic factor in group 2 PH [41,42]. However, the value of the right ventricular dimension as a factor of resistance to sildenafil treatment in patients with end-stage HF and increased PVR has not been sufficiently explored. Initially, at an early stage of PH, the right ventricle adapts to the afterload by increasing wall thickness (hypertrophy, cardiac muscle thickness) and displaying contractility to maintain the flow [43,44]. In the case of a progressive increase in PVR observed in the left-sided PH, the right ventricle remodels from a low-pressure to a high-pressure pump. Over the time, the right ventricular function ceases to match the higher afterload, while the right ventricle gradually enlarges to limit the reduction in stroke volume [44,45]. As a result that the right ventricle adapts to the increasing vascular load through gradual hypertrophy lasting until the end stage of the disease, when progressive dilation begins, the right ventricular dimension seems an essential parameter to predict the outcomes in patients with PH [43,44,45,46]. Thus, the increasing dimension of the right ventricle reflects the progress of left-sided PH and—indirectly—the lack of efficacy of sildenafil treatment as an expression of profound changes in the pulmonary bed.

There are some important limitations to the present study, primarily a relatively small study population with a limited sample size, which calls for the results to be interpreted with caution. Prospective and multicenter studies with a large number of patients are required to clarify the associations between our independent factors and the lack of response to sildenafil therapy in some patients with end-stage HF and elevated PVR. Future studies should explore the mechanisms that might underlie the relationship of the above factors with the ineffectiveness of sildenafil treatment, which would eventually help us identify the patients who do not respond to the treatment with sildenafil. This is of utmost importance, given the possible side effects of sildenafil in a long-term follow-up and the lack of benefit from sildenafil therapy in some patients with reversible PVR. In addition, in our study echocardiographic assessment of the right ventricle was limited to the RVEDd and the tricuspid annular plane systolic excursion (TAPSE).

## 5. Conclusions

To the best of our knowledge, the present study is the first report to demonstrate that higher concentrations of TNC, fibrinogen and creatinine, as well as a larger RVEDd are independently associated with the ineffectiveness of sildenafil treatment in patients with elevated PVR during a six-month follow-up. Among the independent risk factors of the resistance to sildenafil treatment, TNC has the strongest predictive power, sensitivity and specificity, allowing for an effective separation of patients who respond to sildenafil therapy from those who do not. In addition, our study provides simple and non-invasive markers that can help identify patients who will benefit from sildenafil therapy

## Figures and Tables

**Table 1:** Baseline characteristics of the study population.

Parameters	All Included N = 132 ^c^	Group A ^a^ N = 83	Group B ^b^ N = 49	*p* ^d^
Basic parameters				
Age, years	57.5 (50.5–62.00)	57.00 (50.00–61.00)	58.00 (51.00–62.00)	0.31
Male, *n* (%)	119 (90.2)	72 (86,7)	47 (95.9)	0.08
NYHA IV, *n* (%)	89 (67.4)	53 (63.9)	36 (73.5)	0.25
NYHA III, *n* (%)	43 (32.6)	30 (36.1)	13 (26.5)	
Ischemic etiology of HF, *n* (%)	92 (69.7)	56 (67.5)	36 (73.5)	0.28
BMI, kg/m^2^	26.44 (24.69–30.00)	26.12 (24.69–29.26)	27.16 (24.76–31.20)	0.32
Comorbidities				
Hypertension, *n* (%)	64 (48.5)	41 (49.4)	23 (46.9)	0.78
Type 2 diabetes, *n* (%)	57 (43.2)	39 (47)	18 (36.4)	0.25
*Persistent* FA, *n* (%)	64 (49.5)	43 (51.8)	25 (51)	0.93
Laboratory parameters				
Hemoglobin, mmol/L	8.30 (7.50–9.10)	8.30 (7.50–9.10)	8.40 (7.70–9.20)	0.62
Creatinine, µmol/L	117.5 (101.5–138.00)	114.50 (99.00–136.00)	126.00 (107.00–153.00)	0.052
Total bilirubin, µmol/L	24.50 (16.05–34.85)	18.50 (13.00–24.60)	38.50 (29.40–44.60)	<0.001 ^d^
Albumin, g/L	42.00 (40.00–44.00)	41.22 (4.52)	42.49 (3.54)	0.09
Uric acid, µmol/L	457.00 (383.00–585.00)	438.00 (381.00–521.00)	567.00 (420.00–640.00)	0.007 ^d^
Urea, µmol/L	9.70 (6.80–13.50)	9.40 (6.70–13.00)	10.95 (6.80–16.05)	0.19
Sodium, mmol/L	138 (134.0–140.0)	137.65 (4.63)	137.37 (4.93)	0.74
Fibrinogen, mg/dL	396.00 (337.00–397.00)	354.00 (313.00–419.00)	478.00 (416.00–531.00)	<0.001 ^d^
AST, U/L	24.50 (20.00–31.00)	26.00 (20.00–37.00)	23.00 (19.00–28.00)	0.03
ALT, U/L	20.50 (16.00–30.00)	23.00 (16.00–34.00)	20.00 (17.00–24.00)	0.08
ALP, U/L	99.00 (76.00–126.00)	93.50 (70.00–123.00)	112.00 (89.00–133.00)	0.008 ^d^
GGTP, U/L	134 (86.00–182.00)	98.00 (65.00–139.00)	201.00 (153.00–244.00)	<0.001 ^d^
Cholesterol, mmol/L	3.91 (3.00–4.67)	4.03 (2.96–4.74)	3.81 (3.04–4.50)	0.26
hs-CRP, mg/L	7.2 (3.4–12.50)	4.31 (2.38–8.16)	14.27 (11.10–21.33)	<0.001 ^d^
NT-proBNP, pg/mL	3501 (2271–5736.5)	3273 (1901–5670)	4302(3000–6101)	0.07
Tenascin C, ng/L	756.299 (357.96–2489.29)	446.26 (185.36–678.92)	3286.35 (1250.42–4312.33)	<0.001 ^d^
Echocardiografic parameters				
RVEDd, mm	40.00 (32.00–43.00)	34.00 (31.00–41.00)	42.00 (40.00–45.00)	<0.001 ^d^
TAPSE, mm	14 (13–16)	15 (13–16)	14 (12–15)	0.06
LVEDd, mm	75.72 (9.13)	75.52 (9.67)	76.06 (8.23)	0.72
LA, mm	55.00 (50.0–60.00)	54.00 (49.00–60.00)	58.00 (54.00–60.00)	0.03 ^d^
LVEF, %	16.00 (15.00–20.00)	18.00 (15.00–20.00)	16.00 (15.00–19.00)	0.27
Treatment				
B-blockers, *n* (%)	126 (95.5)	78 (94.0)	48 (98.0)	0.41
ACEI/ARB, *n* (%)	99 (80.5)	66 (79.5)	41 (83.7)	0.65
Loop diuretics, *n* (%)	130 (98.5)	82 (98.8)	48 (98.0)	0.70
MRA, *n* (%)	130 (98.5)	82 (98.8)	48 (98.0)	0.70
Digoxin, *n* (%)	37 (28.0)	23 (27.7)	14 (28.6)	0.92
Ivabradine, *n* (%)	30 (22.7)	19 (22.9)	11 (22.4)	0.95
Amiodaron, *n* (%)	37 (28.0)	25 (30.1)	12 (24.5)	0.49
Statin, *n* (%)	88 (66.7)	55 (66.3)	33 (67.3)	0.90
Coumarin derivatives, *n* (%)	99 (75.0)	64 (77.1)	35 (71.4)	0.47
ICD, *n* (%)	71 (53.8)	46 (55.4)	25 (51)	0.62
CRT-D, *n* (%)	59 (44.7)	35 (42.2)	24 (49)	0.45
Other				
VO_2_ max, mL/kg/min, median (IQR)	12.00 (10.50–13.50)	12.20 (10.60–13.40)	11.40 (9.70–13.60)	0.39

^a^ Group A: patients with PVR < 3 Wood units after 6 months of sildenafil therapy. ^b^ Group B: patients with PVR > 3 Wood units after 6 months of sildenafil therapy. ^c^ Data are presented as medians (25th–75th percentile), means (standard deviation) or number (percentage) of patients. ^d^ *p* < 0.05 (statistically significant). Abbreviations: ACEI, angiotensin-converting-enzyme inhibitor; ALP, alkaline phosphatase; ALT, alanine aminotransferase; ARB, angiotensin II receptor blocker; AST, aspartate aminotransferase; BMI, body mass index; CRT-D, cardiac resynchronization therapy-defibrillator; FA, atrial fibrillation; GGTP, gamma-glutamyl transpeptidase; HF, heart failure; hs-CRP, high-sensitivity C-reactive protein; ICD, implantable cardioverter-defibrillator; LA, left atrium; LVEDd, left ventricular end-diastolic dimension; LVEF, left ventricular ejection fraction; MRA, mineralocorticoid receptor antagonists; NT-proBNP, N-terminal prohormone of brain natriuretic peptide; NYHA, New York Heart Association; RVEDd, right ventricular end-diastolic dimension; TAPSE, tricuspid annular plane systolic excursion; Vo_2_ max, maximal oxygen uptake.

**Table 2:** Hemodynamic results of right heart catheterization at the baseline and after 6 months of sildenafil therapy in Groups A and B.

Hemodynamic Results of Right Heart Catheterization						
Parameters	Before Sildenafil Therapy			After 6 Months of Sildenafil Therapy		
	Group A ^a^	Group B ^b^	*p*	Group A ^a^	Group B ^b^	*p* ^c^
sPAP, mmHg	56.00 (52.00–60.00) ^d^	57.00 (55.00–62.00)	0.08	43.00 (40.00–46.00)	54.00 (51.00–58.00)	<0.001 ^c^
mPAP, mmHg	41.66 (5.65)	45.14 (3.99)	<0.001 ^c^	32.00 (30.00–35.00.)	43.00 (40.00–47.00)	<0.001 ^c^
CI, l/min/m^2^	1.80 (1.70–1.90)	1.70 (1.60–1.90)	0.02 ^c^	1.96 (1.90–2.10)	1.80 (1.70–1.90)	<0.001 ^c^
TPG, mmHg	18.94 (3.66)	20.61 (3.59)	0.01 ^c^	12.00 (10.00–13.00)	19.00 (16.00–21.00)	<0.001 ^c^
PVR, Wood units	4.40 (4.10–5.25)	5.48 (4.57–6.07)	<0.001 ^c^	1.76 (1.41–2.08)	3.78 (3.35–4.470)	<0.001 ^c^

^a^ Group A: patients with PVR <3 Wood units after 6 months of sildenafil therapy ^b^ Group B: patients with PVR > 3 Wood units after 6 months of sildenafil therapy. ^c^ *p* < 0.05 (statistically significant). ^d^ Data are presented as medians (25th–75th percentile) or means (standard deviation). Abbreviations: CI, cardiac index; mPAP, mean pulmonary artery pressure; sPAP, systolic pulmonary artery pressure; PVR, pulmonary vascular resistance; TPG, transpulmonary gradient.

**Table 3:** Univariable and multivariable factors associated with the ineffectiveness of sildenafil treatment.

Parameters	Univariable Data		Multivariable Data	
	OR	*p* ^a^	OR	*p*
Tenascin C	1.003 (1.002–1.005)	<0.001 ^a^	1.004 (1.002–1.006)	0.0003 ^a^
Albumin	1.077 (0.987–1.176)	0.10		
Total bilirubin	1.192 (1.122–1.266)	<0.001 ^a^		
hs-CRP	1.423 (1.264–1.603)	<0.001 ^a^		
Fibrinogen	1.013 (1.008–1.018)	<0.001 ^a^	1.019 (1.005–1.033)	0.009 ^a^
ALP	1.007 (1.998–1.015)	0.14		
GGTP	1.020 (1.012–1.028)	<0.001 ^a^		
Creatinine	1.012 (1.001–1.023)	0.04 ^a^	1.025 (1.004–1.047)	0.02 ^a^
Uric acid	1.004 (1.001–1.006)	0.005 ^a^		
Urea	1.051 (0.988–1.118)	0.11		
RVEDd	1.146 (1.078–1.219)	<0.001 ^a^	1.279 (1.074–1.525)	0.006 ^a^

^a^*p* < 0.05 (statistically significant). Abbreviations: ALP, alkaline phosphatase; GGTP gamma-glutamyl transpeptidase; hs-CRP, high-sensitivity C-reactive protein; OR, odds ratio; RVEDd, right ventricular end-diastolic dimension.

**Table 4:** A summary of ROC curve analysis.

	AUC (±95 CI)	Cut-off	Sens. (±95 CI)	Spec. (±95 CI)
Tenascin-C	0.9680 (0.9444–0.9916)	>999	0.95 (0.86–0.99)	0.89 (0.80–0.95)
Fibrinogen	0.8187 (0.7456–0.8917)	>401	0.85 (0.73–0.94)	0.77 (0.61–0.82)
RVEDd	0.7577 (0.6723–0.8431)	>40	0.88 (0.75–0.95)	0.65 (0.54–0.75)
Creatinine	0.6025 (0.4981–0.7070)	>153	0.26 (0.15–0.41)	0.95 (0.88–0.99)

Abbreviations: AUC, area under the curve; CI, confidence interval; RVEDd, right ventricular end-diastolic dimension.
